# The HSV-1 ubiquitin ligase ICP0: Modifying the cellular proteome to promote infection

**DOI:** 10.1016/j.virusres.2020.198015

**Published:** 2020-08

**Authors:** Milagros Collados Rodríguez, Joseph M. Dybas, Joseph Hughes, Matthew D. Weitzman, Chris Boutell

**Affiliations:** a MRC-University of Glasgow Centre for Virus Research, Glasgow, Scotland, United Kingdom; b Division of Protective Immunity and Division of Cancer Pathobiology, Children’s Hospital of Philadelphia, Philadelphia, PA, USA; c Department of Biomedical and Health Informatics, Children’s Hospital of Philadelphia, Philadelphia, PA, USA; d Department of Pathology and Laboratory Medicine, University of Pennsylvania Perelman School of Medicine, Philadelphia, PA, USA

**Keywords:** ATM, Ataxia Telangiectasia-Mutated, ATRX, Alpha Thalassemia/mental Retardation syndrome X-linked, CENP, CENtromere Protein, ChIP, Chromatin Immuno Precipitation, Daxx, Death domain Associated protein, ΔICP0, null mutant ICP0 from HSV-1, FHA, ForkHead Associated, HAUSP, Herpesvirus-Associated Ubiquitin-Specific Protease, HIRA, HIstone cell cycle Regulator defective homologue A, IFI16, IFN-γ Inducible protein 16, IFN, InterFeroN, IRF3, Interferon Regulatory Factor 3, MDC1, Mediator of DNA damage Checkpoint 1, MRE11, Meiotic Recombination 11 Homolog 1, NBS1, Nijmegen Breakage Syndrome 1 (Nibrin), NF-κB, Nuclear Factor Kappa B, NLS, nuclear localization Signal, PIAS, Protein Inhibitor of Activated STAT, PML-NBs, ProMyelocytic Leukaemia Nuclear Bodies, PTM, Post-Translational Modification, RING, Really Interesting New Gene, Sp100, Speckled 100 kDa, STAT, Signal Transducer and Activator of Transcription, STING, STimulator of IFN Genes, STUbL, SUMO-Targeted Ubiquitin Ligase, SUMO, Small Ubiquitin MOdifier, vDNA, viral DNA, TERRA, TElomere Repeat-containing RNA, TPP1, TriPeptidyl Peptidase 1, TP53BP1, Tumor Protein P53 Binding Protein 1, HSV-1, ICP0, Ubiquitin, PML-NBs, Chromatin, Immunity

## Abstract

Herpes simplex virus 1 (HSV-1) hijacks ubiquitination machinery to modify the cellular proteome to create an environment permissive for virus replication. HSV-1 encodes its own RING-finger E3 ubiquitin (Ub) ligase, Infected Cell Protein 0 (ICP0), that directly interfaces with component proteins of the Ub pathway to inactivate host immune defences and cellular processes that restrict the progression of HSV-1 infection. Consequently, ICP0 plays a critical role in the infectious cycle of HSV-1 that is required to promote the efficient onset of lytic infection and productive reactivation of viral genomes from latency. This review will describe the current knowledge regarding the biochemical properties and known substrates of ICP0 during HSV-1 infection. We will highlight the gaps in the characterization of ICP0 function and propose future areas of research required to understand fully the biological properties of this important HSV-1 regulatory protein.

## Highlights

• ICP0 is a viral E3 ubiquitin ligase that promotes HSV-1 infection.
• ICP0 interacts with multiple component proteins of the ubiquitin pathway.
• ICP0 disrupts multiple cellular processes activated in response to infection
• ICP0 remodels the SUMO proteome to counteract host immune defences to infection.
• ICP0 is an attractive drug target for the development of antiviral HSV-1 therapeutics.

## 1 Why are herpesviruses important?

Herpesviruses are ubiquitous viral pathogens that cause a variety of clinically important diseases on a global scale, ranging from mild skin sores and rashes to blindness, congenital birth defects, cancer, and encephalitis (Knipe and Howley, 2013). The reason for their prevalence and evolutionary success is attributable to their ability to enter into a latent state of infection that is maintained for the duration of the host’s lifespan. This latent reservoir of virus evades immune clearance, which can periodically reactivate leading to recurrent disease and transmission to new hosts. Thus, understanding the cellular processes that regulate lytic and latent infection is essential to managing and treating the clinical conditions they cause. Such studies also provide fundamental insight into the molecular mechanisms employed by viruses to evade host immune defences that influence the outcome of infection, offering new opportunities for therapeutic intervention.

## 2 HSV-1 interacts with and hijacks the host ubiquitin machinery

Like many viruses, herpesviruses hijack component proteins of the host ubiquitin (Ub) machinery to subvert cellular processes in order to establish a conducive environment for replication (Isaacson and Ploegh, 2009; Luo, 2016). During HSV-1 infection, these events are largely driven by ICP0, a RING-finger E3 Ub ligase expressed from the outset of nuclear infection (Boutell et al., 2002; Hagglund et al., 2002). While ICP0 is classified as a non-essential viral gene product (Stow and Stow, 1986; Sacks and Schaffer, 1987; Yao and Schaffer, 1995; Chen and Silverstein, 1992), research has established ICP0 to play an important role in modulating the intracellular environment to promote the successful onset of lytic infection and productive reactivation of viral genomes from latency (Stow and Stow, 1986; Sacks and Schaffer, 1987; Yao and Schaffer, 1995; Chen and Silverstein, 1992; Everett et al., 2004; Leib et al., 1989; Halford and Schaffer, 2001; Halford et al., 2001; Thompson and Sawtell, 2006; Cai et al., 1993). Importantly, the requirement for ICP0 during HSV-1 infection is cell type dependent, with many carcinoma cell lines being permissive to HSV-1 ΔICP0 mutant infection relative to normal diploid cells (Stow and Stow, 1986; Yao and Schaffer, 1995; Everett et al., 2004; Alandijany et al., 2018). With respect to osteosarcoma (U2OS and SAOS) cells, permissivity correlates with a defect in the recruitment of key antiviral host factors to infecting vDNA from the outset of nuclear infection (discussed below; (Yao and Schaffer, 1995; Alandijany et al., 2018)). Such differences highlight the restrictive nature of the intrinsic (pre-existing) proteome to the initiation of HSV-1 replication under low MOI conditions that can vary between infected cells (Drayman et al., 2017; Drayman et al., 2019; Cohen and Kobiler, 2016). Thus, understanding the biochemical and biological properties of ICP0 provides valuable insight into host factors and cellular processes which influence the restriction of many viral pathogens.

**Fig. 1:**
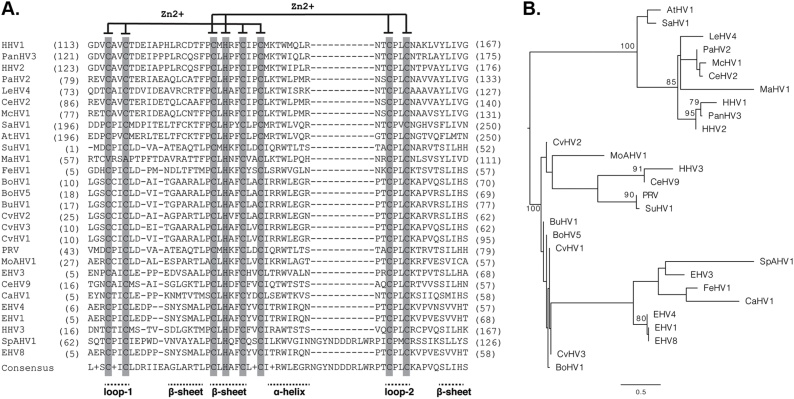
The RING-finger domain of ICP0 is conserved between α-herpesvirus orthologues. A) Alignment of α-herpesvirus (α-HV) ICP0 RING finger domains. Amino acid (aa) residues coordinating the two zinc cations (Zn^2+^) are highlighted in grey. Regions of secondary structure are underlined with horizontal dashes. B) Maximum likelihood phylogeny of the α-herpesvirus RING domain generated using RAxML with the LG amino acid substitution model. The phylogeny is rooted in the middle of the tree (mid-point rooted) and support for the relationships are shown at the nodes of the phylogeny using bootstrap. Only nodes with bootstrap support above 70% are shown. Abbreviation (common name), virus name, protein accession number, RING domain region: HHV1 (HSV-1), Human α-HV 1, YP_009137074.1, 113:167; PanHV3, Chimpanzee herpesvirus strain 105640, YP_009010986.1, 121:175; HHV2 (HSV-2), Human α-HV 2, YP_009137210.1, 123:176; PaHV2, Papiine α-HV 2, YP_443846.2, 79:133; LeHV4, Leporid α-HV 4, YP_009230192.1, 73:127; CeHV2, Cercopithecine α-HV 2, YP_164442.2, 86:140; McHV1, Macacine α-HV 1, NP_851859.2, 77:131; SaHV1, Saimiriine α-HV 1, YP_003933840.1, 196:250; AtHV1, Ateline α-HV 1, YP_009361938.1, 196:250; SuHV1, Suid α-HV 1, YP_068377.2, 1:52; MaHV1, Macropodid α-HV 1, YP_009227214.1, 57:111; FeHV1, Felid α-HV 1, YP_003331582.1, 5:57; BoHV1, Bovine α-HV 1, NP_045363.1, 10:62; BoHV5, Bovine α-HV 5, NP_954951.1, 18:70; BuHV1, Bubaline α-HV 1, YP_0096646811, 17:69; CvHV2, Cervid α-HV 2, AVT50781.1, 25:77; CvHV3, Cervid α-HV 3, AVT50645.1, 10:62; CvHV1, Cervid α-HV 1, AVT50711.1, 10:62; PRV, Pseudorabies virus Ea, AAG17904.1, 43:95; MoAHV1, Beluga whale α-HV 1, ASW27104.1, 27:79; EHV3, Equid α-HV 3, YP_009054966.1, 5:57; CeHV9, Cercopithecine α-HV 9, NP_077475.1, 16:68; CaHV1, Canid α-HV 1, YP_009252287.1, 5:57; EHV4, Equid α-HV 4, NP_045280.1, 6:58; EHV1, Equid α-HV 1, YP_053107.1, 5:57; HHV3 (VZV), Human α-HV 3, NP_040183.1, 16:68; SpAHV1, Sphenicid α-HV 1, YP_009342410.1, 62:126; EHV8, Equid α-HV 8, YP_006273043.1, 5:58.

**Fig. 2:**
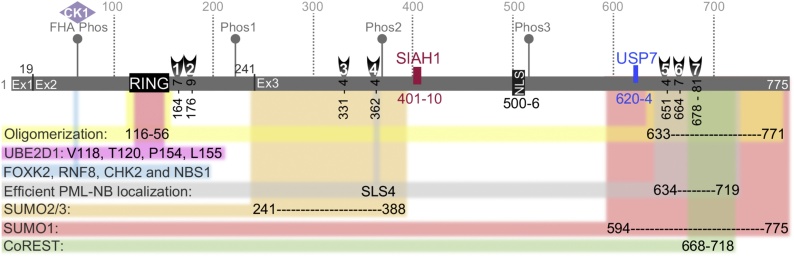
ICP0 RING-finger dependent and independent interactions. Schematic representation of the ICP0 ORF amino acid (aa) residues 1-775. Spliced exons (Ex): Ex1, aa 1-19; Ex2, aa 20-241; Ex3, aa 242-775. Ex2 harbours the RING finger domain (black box, aa 116-156) influencing ICP0 oligomerization together with aa 633-771 (highlighted yellow; (Meredith et al., 1995; Ciufo et al., 1994; Mullen et al., 1994)). ICP0 RING-finger residues required for UBE2D1 interaction (highlighted fuchsia (Vanni et al., 2012)). ICP0 phosphorylation motifs are indicated with grey pins: FHA domain (pS64 and 67-pTELF-70; (Chaurushiya et al., 2012)) recruit CK1 (lilac rhombus), FOXK2, and DDR proteins RNF8, CHK2 and NBS1 (highlighted blue; (Chaurushiya et al., 2012)). ICP0 phosphorylation regions Phos1 (S224, T226, T231, T232); Phos2 (S365, S367, S371), and Phos3 (S508, S514, S517, T518) (Davido et al., 2005). SLSs 1-7 (aa 164-167, 176-179, 331-334, 362-363, 651-654, 664-667 and 678-681, respectively) are indicated with black vertical half-moons (Boutell et al., 2011); SLS4 and aa 634-719 influence PML-NB localization (highlighted grey; (Everett et al., 2014)). SIAH binding motif (aa 401-410; (Czechowicz et al., 2018)), a NLS (aa 500-506), and USP7 binding motif (aa 620-624) are indicated with vertical rectangles. ICP0 regions of SUMO2/3 (aa 241-388, highlighted orange) and SUMO1 (aa 594-775, highlighted red) binding shown (Boutell et al., 2011; Everett et al., 2014). CoREST interaction region (aa 668-718, highlighted green; (Gu and Roizman, 2007)).

**Fig. 3:**
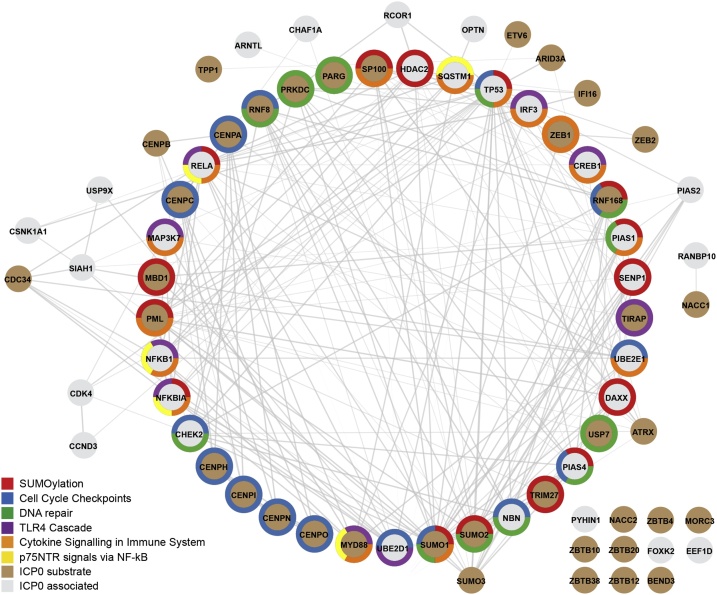
ICP0 interacts with a wide variety of overlapping host pathways. Protein interaction network of host proteins reported to associate with (grey inner circles) or to be degraded (brown inner circles) by ICP0 during HSV-1 infection. Proteins identified by manual curation of the literature. Interaction network and functional analysis was generated using STRING (high confidence threshold, 0.7). Pathway annotations based on the Reactome database. Proteins enriched in the top 6 pathways are coloured (Outer circle, as indicated). Proteins that show interconnectivity are highlighted by grey lines.

Ubiquitination of proteins occurs in a sequential cascade consisting of E1 (Ub activating, UBE1), E2 (Ub conjugating, UBE2), and E3 (Ub ligating) enzymes (Pohl and Dikic, 2019). The Ub ligase activity of ICP0 is entirely attributable to its N-terminal C3HC4 Zn^2+^-binding RING-finger (residues 116-156), a structural domain conserved between α-herpesvirus ICP0 orthologues (Fig. 1; (Boutell et al., 2002; Everett et al., 1993; Everett et al., 1995; Everett et al., 2010; Grant et al., 2012; Lium and Silverstein, 1997)). ICP0 interacts directly with UBE2D1-4 (UbcH5a-d) and UBE2E1-3 (UbcH6a-c) Ub conjugating enzymes (7 out of 37 known human UBE2 proteins; (Boutell et al., 2002; Gu and Roizman, 2003; Vanni et al., 2012; Michelle et al., 2009)) via amino acid (aa) residues located on loop-1, loop-2, and α-helix of its RING-finger domain (Fig. 1, Fig. 2; (Vanni et al., 2012)). ICP0 facilitates the transfer of Ub from charged UBE2 enzymes onto lysine (K) residues within target-substrate(s) with which it interacts (Everett, 2000; Boutell et al., 2003). As Ub contains seven K residues (K6, K11, K27, K29, K33, K48, and K63), anchored Ub molecules can be further ubiquitinated to form linear or branched poly-ubiquitin chains (Ohtake and Tsuchiya, 2017). Proteins conjugated with K48-linked chains are generally considered to be preferential substrates for proteasomal degradation (Braten et al., 2016; Nandi et al., 2006; Chen et al., 2016). UBE2D1-4 and UBE2E1-3 conjugating enzymes are highly conserved among Eukaryotes and known to support the K48-linked poly-ubiquitination of a wide range of substrates (Michelle et al., 2009). Accordingly, α-herpesvirus ICP0 family members have been shown to share similar biochemical properties in the presence of these UBE2 enzymes (Everett et al., 2010). However, non-primate orthologues fail to complement fully the replication defect of an HSV-1 ΔICP0 mutant, indicative of species-specific mechanisms of Ub ligase substrate targeting ((Everett et al., 1995; Everett et al., 2010); reviewed in (Boutell and Everett, 2013)). Importantly, mutation of ICP0-UBE2 interaction residues inactivate ICP0 function (Grant et al., 2012; Lium and Silverstein, 1997; Vanni et al., 2012), highlighting the importance of these host interactions in the biological lifecycle of HSV-1. As such, ICP0 represents an attractive drug target for the development of antiviral HSV-1 therapeutics (Grant et al., 2012; Deschamps et al., 2019; Boutell and Davido, 2015). It remains to be determined if ICP0 can interact with other UBE2 enzymes that may facilitate the differential ubiquitination of substrates or formation of alternative chain types, for example N-terminal methionine-linked (Met1-linked) or K63-linked poly-ubiquitin chains (Griffiths et al., 2013; Metzger et al., 2014). ICP0 can also interact with a variety of cellular E3 Ub ligases, including RNF8, RNF168, SIAH1, and TRIM27 (Fig. 3) (Lilley et al., 2010; Chaurushiya et al., 2012; Nagel et al., 2011; Conwell et al., 2015). It remains to be examined if these interactions can influence the biochemical properties of ICP0 or extend its repertoire of substrates independently of their respective degradation (discussed below; Fig. 3).

Ubiquitinated substrates can be recognized by deubiquitinase (DUB) enzymes, which cleave Ub chains from substrates (Mevissen and Komander, 2017). ICP0 can induce its own autoubiquitination leading to its proteasomal degradation (Boutell et al., 2002; Vanni et al., 2012; Canning et al., 2004). ICP0 counteracts this process by recruiting USP7 (previously known as HAUSP (Meredith et al., 1995; Everett et al., 1997); Fig. 2), which cleaves anchored Ub chains from ICP0 leading to its stabilization (Canning et al., 2004; Everett et al., 1999a). Reciprocally, ICP0 can ubiquitinate USP7 leading to its proteasomal degradation in an ICP0 phosphorylation-dependent manner (Boutell et al., 2005; Mostafa et al., 2013). Recent structural studies have solved the interaction interface between ICP0 and USP7 which has been proposed as a potential drug target (Pozhidaeva et al., 2015; Pfoh et al., 2015). It remains to be established whether ICP0’s interaction with USP7 can lead to the stabilization of other ICP0 interaction partners or influence Ub chain editing to promote substrate degradation. ICP0 can also interact with USP9X (Sato et al., 2016), although the biochemical relationship between these two proteins remains to be investigated. The HSV-1 deubiquitinase enzyme UL36 has also been reported to influence ICP0 expression levels during infection (Kattenhorn et al., 2005; Wang et al., 2013), although it remains to be determined if UL36 can catalyse the deubiquitination of ICP0 directly. Collectively, these observations demonstrate ICP0 to interact with multiple component enzymes of the Ub pathway to promote a conducive environment favourable to HSV-1 replication.

## 3 ICP0-mediated degradation of PML-NBs and SUMOylated host proteins

ICP0 was initially identified as an E3 Ub ligase by virtue of its ability to localize to and disrupt PML-NBs (Everett, 2000; Maul et al., 1993; Maul and Everett, 1994; Everett and Maul, 1994; Everett et al., 1998; Chelbi-Alix and de The, 1999). PML-NBs are highly dynamic nuclear substructures composed of more than 70 proteins that respond to a variety of stimuli, including heat shock, cytokine signalling, and virus infection (Lang et al., 2010; Van Damme et al., 2010; Hoischen et al., 2018; Lang et al., 2019; Bernardi and Pandolfi, 2007; Maul et al., 1995). Infecting HSV-1 genomes are rapidly entrapped by PML-NBs upon nuclear entry (Alandijany et al., 2018; Dembowski and Deluca, 2017), a host response that can lead to viral genome silencing as a component of the intrinsic antiviral immune response (discussed below) (Alandijany et al., 2018; Everett et al., 2006; Everett et al., 2008a; Glass and Everett, 2013; Cabral et al., 2018). PML, the main scaffolding protein of PML-NBs (Ishov et al., 1999), and Sp100 were among the first substrates identified to be degraded by ICP0 in a RING-finger dependent manner (Fig. 3) (Everett et al., 1998; Everett et al., 2009). Consequently, the biological role of PML-NBs during virus infection has been extensively studied, which has revealed these discrete nuclear substructures to play an important function in the spatiotemporal regulation of host immune defences to virus infection (Alandijany et al., 2018; McFarlane et al., 2019; Geoffroy and Chelbi-Alix, 2011).

The post-translational modification of proteins with SUMO plays a key role in the assembly of PML-NBs via a network of protein-protein interactions mediated between constitutively SUMOylated proteins (e.g. PML and Sp100) and resident proteins that contain SUMO Interaction Motifs (SIMs) (Ishov et al., 1999; Muller et al., 1998; Zhong et al., 2000; Shen et al., 2006; Lin et al., 2006). Inhibiting cellular ubiquitination enriches SUMO-modified transcription factors and DNA repair proteins at PML-NBs (Sha et al., 2019), highlighting the dynamic composition of PML-NBs. ICP0 contains seven SIM-Like Sequences (SLSs; Fig. 2) and shares biochemical properties similar to that of cellular SUMO Targeted Ubiquitin Ligases (STUbLs) (Everett et al., 1998; Chelbi-Alix and de The, 1999; Boutell et al., 2003; Boutell et al., 2011; Cuchet-Lourenco et al., 2012; Sloan et al., 2015). ICP0 utilizes a combination of SUMO-dependent and -independent targeting strategies to ubiquitinate and degrade PML isoforms I-VI and Sp100 leading to the disruption of PML-NBs and release of viral genomes entrapped therein (Alandijany et al., 2018; Everett et al., 2009; Boutell et al., 2003; Boutell et al., 2011; Cuchet-Lourenco et al., 2012; Everett et al., 2014). Of note, the structure of ICP0 SLS4 (residues 362-367; Fig. 2) and SUMO has recently been solved by NMR (Hembram et al., 2020), revealing cooperation between ICP0 phosphorylation domains (FHA [67-pTELF-70] and Phos2; Fig. 2) in the degradation of SUMOylated proteins (Mostafa et al., 2013; Hembram et al., 2020; Boutell et al., 2008). Importantly, this mechanism of SUMO targeting is not limited to PML-NBs, as ICP0 has been shown to induce the degradation of numerous (≥ 120) SUMOylated proteins including ARID3A/E2FBP1, BEND3, ETV6, MBD1, NACC1, NACC2, ZBTB4, ZBTB10, ZBTB38, and MORC3 (Fig. 3) (Everett et al., 1998; Chelbi-Alix and de The, 1999; Boutell et al., 2011; Sloan et al., 2015). Degradation of MORC3 by ICP0 has been proposed to inhibit the recruitment of PML-NB host factors to infecting viral genomes (Sloan et al., 2016), an observation that warrants further investigation due to the importance of PML-NBs in the intracellular restriction of viral pathogens (Geoffroy and Chelbi-Alix, 2011; Komatsu et al., 2016). In most cases, however, it remains to be determined if the degradation of these SUMOylated proteins is functionally relevant to HSV-1 infection or a consequence of collateral damage by the targeting mechanism employed by ICP0 to disrupt PML-NBs. Notably, it is becoming clear that PML-NB host factors cooperate with host SUMOylation machinery (e.g. PIAS SUMO E3 ligases) to restrict HSV-1 infection (Boutell et al., 2011; Conn et al., 2016; Brown et al., 2016). Depletion of PIAS1 and PIAS4 in combination with PML significantly alleviates the restriction of an HSV-1 ΔICP0 mutant relative to PML depletion alone (Conn et al., 2016; Brown et al., 2016). Thus, ICP0’s ability to disrupt SUMO-SIM interactions through multiple targeting mechanisms is likely to be a common strategy employed by ICP0 to remodel proteome interaction networks that facilitate or maintain the intracellular restriction of HSV-1 (Everett and Murray, 2005; Cuchet-Lourenco et al., 2011; Maroui et al., 2018).

## 4 Interplay between ICP0, chromatin remodelling, and intrinsic immunity

HSV-1 genomes are delivered to the nucleus of newly infected cells as linear molecules of naked DNA (Kilcher and Mercer, 2015), which immediately bind constitutively expressed host factors with pro- and antiviral cellular functions (Dembowski and DeLuca, 2018). ChIP experiments have demonstrated infecting viral genomes to rapidly associate with cellular histones, which can carry distinct epigenetic signatures that influence the progression of viral transcription (Cabral et al., 2018; Cliffe and Knipe, 2008; Kutluay and Triezenberg, 2009; Placek et al., 2009; Merkl et al., 2018; Kristie, 2015; Suzich and Cliffe, 2018). Relevant to ICP0, microscopy experiments have shown the histone H3.3 chaperone complex Daxx/ATRX and IFI16 to associate with infecting HSV-1 genomes prior to vDNA entrapment within PML-NBs (Alandijany et al., 2018; Cabral et al., 2018; Everett, 2015; Diner et al., 2016), known repositories of non-nucleosomal histone H3.3 (Delbarre et al., 2013; Corpet et al., 2014; Drane et al., 2010; Cohen et al., 2018). The recruitment of these host factors correlates with the epigenetic modification of histone H3 (H3K9me3 and H3K27me3) on viral chromatin which can lead to transcriptional silencing in the absence of ICP0 (Cabral et al., 2018; Cohen et al., 2018; Lee et al., 2016). HSV-1 mutants that fail to express ICP0, or carry mutations that abolish its Ub ligase activity, have a significantly lower probability of initiating a productive infection in restrictive cell types (approximately 1000-fold relative to WT HSV-1; (Everett et al., 2004)). Such observations have led to the hypothesis that PML-NBs may act as an axis for vDNA chromatinization and gene silencing (Cohen et al., 2018; Newhart et al., 2012), as vDNA remains stably entrapped in PML-NBs in the absence of ICP0 under low MOI conditions (Alandijany et al., 2018; Cohen et al., 2018; Everett et al., 2007; Maroui et al., 2016). In support of this, resident PML-NB proteins (PML, Daxx and ATRX) have been shown to act cooperatively with IFI16 to restrict HSV-1 ΔICP0 gene expression that correlates with repressive histone signatures (H3K9me3 and H3K27me3) on viral chromatin (Everett et al., 2008a; Cabral et al., 2018; Merkl et al., 2018; Lee et al., 2016). While Sp100 also contributes to the repression of HSV-1 ΔICP0 gene expression (Everett et al., 2008a; Glass and Everett, 2013; Everett et al., 2009), the influence of Sp100 on the epigenetic regulation of viral chromatin remains to be determined. Thus, rapid chromatinization and epigenetic modification of vDNA upon nuclear entry can restrict the initiation of productive HSV-1 infection. Importantly, this intrinsic host response to vDNA nuclear entry occurs prior to the activation of cytokine-mediated innate immune defences under low genome copy-numbers of infection (see below) (Alandijany et al., 2018; Everett et al., 2008b).

Expression of ICP0 induces the degradation and dispersal of PML-NB associated proteins from vDNA (see Section 3) (Alandijany et al., 2018; Cabral et al., 2018; Everett et al., 2013), which leads to a reduction in histone H3 loading and enhanced levels of histone H3 acetylation on vDNA to promote transcription (Cabral et al., 2018; Cliffe and Knipe, 2008; Lee et al., 2016; Ferenczy et al., 2011). ICP0 also induces the degradation of ATRX and IFI16 (Jurak et al., 2012; Orzalli et al., 2012; Orzalli et al., 2016; Cuchet-Lourenco et al., 2013; Diner et al., 2015). However, the turnover of these proteins occurs with delayed kinetics relative to that of PML degradation (Jurak et al., 2012; Cuchet-Lourenco et al., 2013). Such observations are likely to reflect the sequential degradation of host factors as infection progresses (Merkl and Knipe, 2019), a conclusion consistent with the differential accumulation of cellular factors on vDNA throughout the initiating cycle of HSV-1 infection (Dembowski and Deluca, 2017; Dembowski and DeLuca, 2015). Indeed, recent microscopy studies have identified the histone H3.3 chaperone protein HIRA to restrict HSV-1 infection following the onset of vDNA replication, a host response antagonized by ICP0 through the nuclear dispersal of HIRA (McFarlane et al., 2019). Thus, multiple histone H3.3 chaperone proteins (Daxx/ATRX and HIRA) can restrict the progress of HSV-1 ΔICP0 replication at independent phases of infection.

ICP0 has also been reported to bind CoREST, a component protein of the REST/CoREST/HDAC1,2/LSD1 nuclear repressor complex (Fig. 2, residues 668–718; (Gu et al., 2005; Gu and Roizman, 2007)). ICP0 disrupts HDAC1 binding to CoREST, leading to HDAC1 translocation to the cytoplasm (Gu et al., 2005). This ICP0-mediated action is proposed to block viral chromatin histone deacetylation and thus maintain a transcriptionally active state (Ferenczy et al., 2011; Gu and Roizman, 2007; Gu and Roizman, 2009). However, complementation assays have shown ICP0-CoREST binding mutants to have only a modest impact on the acetylation status of viral chromatin relative to that of a functionally active RING-finger domain (Ferenczy et al., 2011). Of interest, the proposed CoREST binding site lies in a region of C-terminal homology conserved between primate ICP0 orthologues (Everett et al., 2014), which plays a multi-functional role in the biological properties of ICP0, including USP7 binding and PML-NB localization (Fig. 2) (Everett et al., 2009; Everett et al., 2014). Thus, while ICP0 has the potential to influence the epigenetic modification of viral chromatin in a RING-finger independent manner, the general consensus is that ICP0’s Ub ligase activity plays a central role in its ability to transactivate viral gene expression to stimulate the progression of infection.

## 5 ICP0 modulation of the cellular DNA Damage Response (DDR) pathway

Like many DNA viruses, HSV-1 shows an intimate relationship with the DDR pathway which can both positively and negatively influence the outcome of infection (Wilkinson and Weller, 2004; Lilley et al., 2005; Smith and Weller, 2015; Dybas et al., 2018). Upon nuclear infection, HSV-1 induces cellular DNA double strand breaks (DSBs) that are sensed by the MRN complex (MRE11, RAD50, and NBS1) which activates ATM leading to the phosphorylation of histones H2A and H2AX (γH2AX) flanking the chromatin break (Shirata et al., 2005). This stimulates the recruitment of MDC1, that recruits the cellular Ub ligases RNF8 and RNF168 to DSBs which ubiquitinate H2A and γH2AX, a signal that promotes the recruitment of downstream repair proteins (Mailand et al., 2007; Doil et al., 2009). ICP0 targets RNF8 and RNF168 for degradation in a FHA-domain and phosphorylation-dependent manner, abrogating H2A and H2AX ubiquitination that restricts the recruitment of host DDR repair factors (e.g. TP53BP1) (Lilley et al., 2010; Chaurushiya et al., 2012). During HSV-1 ΔICP0 mutant infection, recruitment of these DDR proteins occurs in close proximity to vDNA in a PML and Sp100 independent manner (Lilley et al., 2011). Depletion of RNF8 and RNF168 partially relieves the replication defect of an HSV-1 ΔICP0 mutant, demonstrating that RNF8 and RNF168 contribute to the intrinsic antiviral restriction of HSV-1 through a mechanism antagonized by the Ub ligase activity of ICP0. Notably, SUMOylation is also known to play a key role in mediating the recruitment of DDR proteins to DSBs through SUMO-SIM interactions catalysed by PIAS SUMO ligases (Morris et al., 2009; Galanty et al., 2009). Thus, it is likely that ICP0 utilizes SUMO-dependent and -independent targeting strategies to modulate the DDR during HSV-1 infection (Conn et al., 2016; Brown et al., 2016).

DSBs can also be sensed by Ku70/Ku80, leading to the recruitment of DNA-PK which promotes non-homologous end joining (NHEJ) at DSBs. ICP0 has been shown to induce the degradation of the catalytic subunit of DNA-PK (DNA-PKcs/PRKDC; (Lees-Miller et al., 1996; Parkinson et al., 1999)). While it is clear that DNA-PKcs contributes to the cellular restriction of an HSV-1 ΔICP0 mutant, the precise mechanism of restriction remains to be defined but has been linked to viral genome circularization and regulation of innate immune defences (see below) (Jackson and DeLuca, 2003; Burleigh et al., 2020).

ICP0 also localizes to centromeres where it induces the degradation of CENP-A, CENP-B and, CENP-C (Lomonte et al., 2001; Lomonte and Morency, 2007; Everett et al., 1999b), leading to cell cycle arrest and induction of an interphase Centromere Damage Response (iCDR) (Gross et al., 2012). ICP0 has also been shown to promote remodelling of telomeres through the degradation of TPP1, leading to TERRA activation and enhanced levels of HSV-1 replication (Deng et al., 2014). Thus, ICP0’s Ub ligase activity significantly remodels the intracellular chromatin environment to promote the progression of HSV-1 infection.

## 6 ICP0 scrambles innate immune pathways

Detection of viral nucleic acid by host pattern recognition receptors (PRRs) plays a critical role in the activation of signalling cascades that lead to the production of pro-inflammatory cytokines, including type-I, II, and III IFNs (Unterholzner and Almine, 2019; Stempel et al., 2019; Alandijany, 2019). Secretion of IFNs leads to the induction of hundreds of IFN stimulated genes (ISGs) that generate a cellular antiviral state that limits virus propagation and spread. HSV-1 ΔICP0 mutants are hypersensitive to IFN (Everett et al., 2004; Leib et al., 1999; Mossman et al., 2000; Harle et al., 2002), highlighting a role for ICP0 in the regulation of innate immune defences to HSV-1 infection. ICP0 contributes to nuclear PRR inactivation through the degradation of IFI16 and DNA-PKcs, which signal through STING-dependent and -independent pathways, respectively (Diner et al., 2016; Orzalli et al., 2012; Orzalli et al., 2016; Cuchet-Lourenco et al., 2013; Burleigh et al., 2020; Orzalli et al., 2015). Under low MOI conditions vDNA entry into the nucleus alone is not sufficient to trigger PRR activation leading to IFN and ISG expression, which has been shown to require the onset of vDNA replication (Alandijany et al., 2018). PRR sensing by IFI16 during HSV-1 ΔICP0 infection correlates with IFI16 forming nuclear filaments on vDNA in association with PML following the saturation of PML-NBs under high genome loads (Alandijany et al., 2018; Cuchet-Lourenco et al., 2013; Merkl and Knipe, 2019). Such observations highlight a clear segregation in the regulation of intrinsic and innate immune defences that concurrently restrict the initiation and propagation of HSV-1, respectively (Alandijany et al., 2018; Cabral et al., 2018; Everett et al., 2008b). The relative spatiotemporal kinetics of IFI16 and DNA-PKcs PRR sensing of vDNA remains to be determined. Notably, PML isoforms II and IV have been shown to facilitate the loading of transcription factors (including IRF3, NF-κB, and STAT1) onto cellular gene promoters that directly influence the induction of cytokines and ISG expression that contribute to the IFN hypersensitivity of HSV-1 ΔICP0 mutants (McFarlane et al., 2019; Chen et al., 2015; Chee et al., 2003; El Asmi et al., 2014). Thus, ICP0 inactivates intrinsic and innate immune defences early in the infectious cycle through the degradation of PML and cellular PRRs. ICP0 has also been reported to influence the regulation of NF-κB signalling cascades (van Lint et al., 2010; Daubeuf et al., 2009; Zhang et al., 2013). However, the spatiotemporal regulation of this important signalling pathway under physiological infection conditions remains to be fully defined. It also remains to be determined as to what extent ICP0 reshapes the intracellular proteome upon infection of cytokine stimulated cells which express a full complement of ISG products.

## 7 ICP0 and HSV-1 latency

While significant progress has been made in defining the biochemical properties and cellular substrates of ICP0 during HSV-1 lytic infection (Fig. 3), significantly less is known about the Ub ligase activity of ICP0 during viral reactivation from latency. Following primary infection, HSV-1 infects the neuronal dendrites of sensory ganglia that innervate infected tissues. Retrograde transport carries viral capsids along neuronal axons to the nucleus, wherein the viral genome undergoes epigenetic silencing leading to the establishment of latency (for detailed reviews see (Wilson and Mohr, 2012; Nicoll et al., 2012; Bloom, 2016; Cliffe and Wilson, 2017)). With respect to ICP0, animal models have shown ICP0 to be dispensable for the establishment and maintenance of HSV-1 latency, but to play a critical role during viral reactivation leading to *de novo* virus production (Leib et al., 1989; Halford and Schaffer, 2001; Thompson and Sawtell, 2006; Cai et al., 1993). This process occurs in an ICP0 RING-finger and phosphorylation dependent manner (Thompson and Sawtell, 2006; Vanni et al., 2012; Mostafa et al., 2013; Mostafa et al., 2011), highlighting the importance of cellular ubiquitin machinery and kinases in the successful reactivation of HSV-1 from latency. Latent viral genomes can be observed to colocalize at distinct neuronal cell body sub-structures, including *de novo* assembled PML-NBs and centromeres (Cohen et al., 2018; Maroui et al., 2016; Catez et al., 2012), known substrates of ICP0 in mitotic cells (Fig. 3). While it’s tempting to speculate that ICP0 is required to disrupt these nuclear sub-domains that may otherwise promote or maintain vDNA in a state of transcriptional quiescence (Cohen et al., 2018), a number of key observations have been reported that remain to be resolved. Firstly, low levels of ICP0 transcription have been detected in latently infected neurones (Maillet et al., 2006; Chen et al., 2002), although it remains to be determined if ICP0 is expressed or functionally active as a Ub ligase during latency. Secondly, viral reactivation is known to occur in distinct phases; widespread reanimation of non-canonical patterns of gene expression independently of viral protein synthesis (phase-1), followed by sequential patterns of canonical gene expression driven by the transactivating protein VP16 (phase-2) (Kim et al., 2012). ICP0 is required for phase-2 reactivation in a VP16-dependent manner (Thompson and Sawtell, 2006; Thompson et al., 2009). These data indicate that phase-1 reanimation of viral transcription occurs independently of ICP0, a process that has been linked to neuronal stress and DDR pathways (Cliffe et al., 2015; Cliffe, 2019; Hu et al., 2019). Collectively, these observations raise the possibility that ICP0 may have neuronal specific functions or spatiotemporal activities out with of those identified during the initiation of lytic infection in mitotic cells. With the advent of modern cytology techniques that enable the explant or differentiation of neurones *in vitro* (Suzich and Cliffe, 2018; Pourchet et al., 2017; Thellman and Triezenberg, 2017; Thellman et al., 2017; D’Aiuto et al., 2019; Edwards and Bloom, 2019), the molecular function of ICP0 as a viral Ub ligase in modulating neuronal-specific processes, including host immune defences, epigenetic regulation, and the DDR, during HSV-1 latency and reactivation can now be addressed in detail.

## 8 Future Directions: Identification of new ICP0 substrates and host responses to viral infection

While it is clear that the Ub ligase activity of ICP0 plays a central role in the infectious cycle of HSV-1, the full repertoire of ICP0 substrates remains poorly defined. With the development of modern proteomic and bioinformatic methodologies, it is now possible to quantify the impact of ICP0 ubiquitination on both host and viral proteomes. The development of an antibody that recognizes peptides modified by Ub has heralded a significant advancement in the ability to detect, isolate, and quantify ubiquitinated substrates on a proteome-wide scale by mass spectrometry (Xu et al., 2010; Udeshi et al., 2013). Upon trypsin digestion, Ub is cleaved leaving its C-terminal di-glycine bound to K residues in the modified substrate. This di-glycine remnant can be enriched by antibody affinity capture and analyzed by mass spectrometry to identify changes in the cellular ubiquitinome between sample populations. Comparison of WT to ICP0 RING-finger or ΔICP0 mutant HSV-1 infected cells over time would provide quantitative changes in host and viral ubiquitinomes through different phases of infection. Di-glycine remnant profiling in combination with whole cell proteomics would enable the identification of concomitant changes in protein abundance to that of ubiquitination status, enabling the identification of novel ICP0 substrates, new interfaces of viral host interaction, and fundamental insights into cellular functions of ubiquitination in response to virus infection. We hypothesize that ICP0 will target a variety of substrates for ubiquitination that will have proteasome-dependent and -independent functions. Conversely, we hypothesize the host cells will utilize ubiquitination to promote the activation of host immune defences that lead to the cellular restriction of HSV-1 in the absence of ICP0. The application of such proteomic methodologies will significantly advance our understanding of the requirements for ICP0 to remodel the cellular proteome under a range of conditions pertinent to HSV-1 infection; including cell type (e.g. epithelial *vs*. neuronal origin) and immunological status (e.g. resting *vs*. cytokine stimulated). Such studies will likely reveal new and important insights into cellular processes and host factors that mediate the spatiotemporal regulation of immune defences to HSV-1 infection.

## 9 Conclusion

ICP0 hijacks Ub machinery to disrupt cellular pathways that play important roles in the regulation of host immunity and cellular homeostasis, which to date includes the regulation of PML-NBs and host SUMOylation, epigenetic modification, DDR, and the cell cycle (summarized in Fig. 3). The outcome of this proteome remodelling creates a favourable environment to promote the onset of HSV-1 lytic replication, propagation, and productive reactivation of viral genomes from latency. As such, the identification and development of small molecule inhibitors to ICP0 would provide significant therapeutic application in the treatment of recurrent HSV-1 infections by complementing host immune defences to block viral reactivation from latency.

## Author contributions

MCR, JMD, and CB wrote the original draft. MCR, JMD, and JH prepared the figures. JH performed the bioinformatic analysis. MDW and CB edited the manuscript.

## Funding

This work was supported by the 10.13039/501100007155Medical Research Council (https://mrc.ukri.org) grant MC_UU_12014/5 (to CB) and by grants from the 10.13039/100000002National Institutes of Health (AI115104 and NS082240 to MDW, and AI147587 to JMD). The funders had no role in the study design or preparation of this manuscript.

## Declaration of Competing Interest

The authors declare no conflict of interests.
